# Does menopausal status impact urinary continence outcomes following abdominal sacrocolpopexy without anti-incontinence procedures in continent women?

**DOI:** 10.12669/pjms.324.9928

**Published:** 2016

**Authors:** Abdurrahman Hamdi Inan, Emrah Toz, Emrah Beyan, Tutku Gurbuz, Aykut Ozcan, Oznur Oner

**Affiliations:** 1 Abdurrahman Hamdi Inan, Department of Gynecology and Obstetrics, Ardahan State Hospital, Ardahan, Turkey; 2 Emrah Toz, Department of Gynecology and Obstetrics, Izmir Tepecik Education and Research Hospital, Izmir, Turkey; 3 Emrah Beyan, Department of Gynecology and Obstetrics, Izmir Tepecik Education and Research Hospital, Izmir, Turkey; 4 Tutku Gurbuz, Department of Gynecology and Obstetrics, Ardahan State Hospital, Ardahan, Turkey; 5 Aykut Ozcan, Department of Gynecology and Obstetrics, Izmir Tepecik Education and Research Hospital, Izmir, Turkey; 6 Oznur Oner, Department of Gynecology and Obstetrics, Ardahan Military Hospital, Ardahan, Turkey

**Keywords:** Abdominal sacrocolpopexy, *De novo* stress urinary incontinence, Menopausal status, Pelvic organ prolapse

## Abstract

**Objective:**

We investigated the impact of menopausal status on urinary continence following abdominal sacrocolpopexy (ASC) without an anti-incontinence procedure in continent women.

**Methods:**

We conducted a clinical follow-up study of 137 patients diagnosed with stage 3 or higher pelvic organ prolapse (POP) without urinary incontinence between January 2012 and December 2014. Patients were provided with detailed a priori information pertaining to the abdominal sacrocolpopexy procedure and were invited to attend follow-up visits at 1, 3, 12, and 24 months. Follow-up visits included a gynecological examination, cough test, and validated Urinary Distress Inventory-6 (UDI-6) and Incontinence Impact Questionnaire-7 (IIQ-7) questionnaires.

**Results:**

The mean follow-up time for the cohort was 16.5±3.45 months. The study group was divided according to menopausal status: premenopausal (Group-1) and postmenopausal women (Group-II). Anatomical recurrence was not detected during the follow-up period in either group, but de novo stress urinary incontinence was seen in 15 of 53 (28.3%) Group-I patients and in 6 of 84 (7.1%; p < 0.01) Group-II patients.

**Conclusions:**

The risk of de novo stress urinary incontinence in postmenopausal women after ASC is low. However, premenopausal patients have a higher incidence of de novo stress incontinence which affect quality of life.

## INTRODUCTION

Pelvic organ disorders are major health issues for women, affecting their quality of life and often requiring costly surgery.^1^,^2^ However, the prevalence varies widely across studies because of underreporting and the lack of consistent definitions. Approximately one in every four women will be affected by some form of pelvic floor disorder,^3^ and the estimated lifetime cumulative risk of surgery is 7-11%.^4^

Various surgical techniques for prolapse surgery using laparotomy, laparoscopy, and vaginal surgery, including colporrhaphy (with or without mesh placement), sacrospinous ligament fixation, abdominal sacrocolpopexy (ASC), high sacrouterine ligament fixation, and colpocleisis have been used.^5^

Sacrocolpopexy is considered the gold standard for the repair of vaginal vault prolapse. ASC was first described in 1957 by Lane to treat vaginal vault prolapse after a hysterectomy. Sacrocolpopexy can also be performed laparoscopically or using robotic surgical systems, achieving similar cure rates to those obtained using an open approach.^6^-^8^ However, laparoscopic sacrocolpopexy involves extensive suturing and retroperitoneal dissection, requiring advanced skills, and robotic sacrocolpopexy is expensive, requires special training, and is only available at a few treatment centers. Because of these reasons, in our institute, we can only perform ASC.

In the randomized controlled Colpopexy and Urinary Reduction Efforts (CARE) trial that compared sacrocolpopexy with versus without Burch colposuspension to prevent stress urinary incontinence (SUI), Brubaker et al.^9^ reported cure rates as high as 95% at the vaginal apex in women two years after ASC. They also reported that in women without stress incontinence who were undergoing abdominal sacrocolpopexy for prolapse, Burch colposuspension significantly reduced postoperative symptoms of stress incontinence without increasing other lower urinary tract symptoms. However, Costantini et al.10 reported that Burch colposuspension did not provide any additional benefit in pelvic organ prolapse repair in patients with urinary incontinence.

Oestrogen is of great importance in the supportive mechanism of the pelvis by controlling the synthesis and breakdown of collagen.^11^ Also, the tissues of the female urinary continence mechanism are sensitive to oestrogen. Oestrogens may affect continence by enhancing urethral resistance by increasing the number of periurethral vessels.^12^ Moreover, oestrogens can reduce the frequency and amplitude of detrusor contractions and so raise the sensory threshold of the bladder and promote relaxation of the detrusor muscle.^13^,^14^ For these reasons the decline in available oestrogen is a possible etiological factor for pelvic floor disorders include stress urinary incontinence.

In light of these facts, it is expected that premenopausal women are more sensitive to hormonal fluctuations which will affect the structure and function of the lower urinary tract tissue and cause urinary incontinence. Despite these facts, many studies have been performed to compare the outcomes of ASC with versus without an anti-incontinence procedure; however, these studies did not take into consideration the menopausal status of the women.^7^-^10^

The primary aim of this study was to investigate the impact of menopausal status on urinary continence following ASC without an anti-incontinence procedure in continent women. Secondary aims are; changes in subjective symptoms and quality of life, as measured by the IIQ-7 and UDI-6, and differences in anatomical outcome.

## METHODS

This prospective, observational study was conducted between January 2012 and December 2014. Patients who were referred for stage 3 or higher POP repair were evaluated preoperatively including a detailed history (age, parity, body mass index, and existing co-morbidities), gynecological examination (in the gynecological and standing positions, at rest and under maximum straining with a full bladder) for the presence of pelvic organ prolapse (staged according to the Pelvic Organ Prolapse Quantification system,^15^ transvaginal sonography, and a stress test with prolapse reduction with large cotton swab (with patients placed in both supine and standing position and asked to cough after their bladders were filled with 300 mL saline). The test was considered positive if leakage occurred during coughing. We also performed urinalysis and fasting biochemical analysis (including glucose and calcium levels and liver and renal function) and administered the validated Urinary Distress Inventory-6 (UDI-6) and Incontinence Impact Questionnaire-7 (IIQ-7) questionnaires.^16^,^17^

### Inclusion and Exclusion criteria

Patients with stage 3 or higher pelvic organ prolapse (POP) and without urinary incontinence were included in the study. Exclusion criteria were malignant uterine lesions, previous prolapse or incontinence surgery, diseases known to affect bladder function, known hypersensitivity to synthetic materials (polypropylene), use of exogenous estrogen before or after surgery, urinary incontinence and pregnancy. All patients were informed in detail about the operation and the study. All patients gave informed consent, and the study was approved by the local ethics committee. Primary outcome measure was changes in continence status. Secondary outcomes were changes in subjective symptoms and quality of life, as measured by the IIQ-7 and UDI-6 and the anatomical outcome of prolapse repair.

One hour before surgery, all patients were given 1g cefazolin (Cezol; Deva, Istanbul, Turkey). All surgeries were performed via a Pfannenstiel incision. After performing the hysterectomy, the peritoneum overlying the sacral promontory was incised to expose the pre-sacral ligament. Then, the peritoneum on the right side was incised until the cul-de-sac of Douglas to prepare the space for positioning of the prosthesis. The vaginal vault was dissected to expose the pubo-cervical fascia, the uterosacral/cardinal complex, and the rectovaginal fascia. The mesh was attached with four polyglycolic 1-0 sutures on the anterior vaginal wall. The posterior vaginal wall was freed, and the mesh was attached with four more polyglycolic 1-0 sutures. The two polypropylene prostheses were tailored and placed in the sacral periosteum, approximately 2 cm below the promontory using two non-resorbable 2-0 sutures. The peritoneum was closed over the meshes.

Antibiotic and antithrombotic prophylaxes were administered. Bladder catheterization was achieved using a 16-French Foley catheter, which was routinely removed after 24 hour. All patients were invited to attend follow-up visits at 1, 3, 12, and 24 months post-surgery. Follow-up visits included gynecological examinations involving POP quantification, the cough test, and the validated UDI-6 and IIQ-7 questionnaires. Between January and February 2015, as an updated follow-up, all patients were reevaluated by an independent examiner who was blinded to the patient baseline data to eliminate any bias.

All analyses were performed using the SPSS software (ver. 18 for Windows; SPSS Inc., Chicago, IL, USA). Clinical data are presented as means ± standard deviations and percentages, when appropriate. Comparisons between preoperative and postoperative symptoms, including IIQ-7 and UDI-6 scores, were performed using the Wilcoxon matched pairs test. The Mann-Whitney U-test was used to compare variables for independent data. Categorical data were analyzed by McNemar’s test, Fisher’s exact test, or the χ^2^ test. A p-value < 0.05 was taken to indicate statistical significance.

## RESULTS

Of the 145 patients enrolled, 8 were lost to follow-up. Thus, 137 preoperatively continent patients were included in the study. The study group was divided according to menopausal status: premenopausal women (Group-I) and postmenopausal women (Group-II). No patient had a previous prolapse repair or hysterectomy, and thus all were primary POP cases. The mean age of the participants was 51.00±9.79 (range 33-83) years.

Baseline demographic and clinical characteristics of the two groups are summarized in Table-I. Parity and BMI did not differ significantly between the groups. As anticipated, patients in the premenopausal group were significantly younger than those in the postmenopausal group (p < 0.01).

**Table-I:** Baseline demographic and clinical characteristics of the two groups.

	Premenopausal (n = 53)	Postmenopausal (n = 84)	P value
Mean age (year) ± SD (range)	44.29±4.05 (33-51)	57.37±9.39 (43-83)	< 0.01^a^
Mean parity	4.34±1.87 (0-10)	4.95±2.63 (0-12)	0.11^a^
Mean body mass index (kg/m2)	27.2±5.7 (22-32.7)	29.1 ±3.1 (23.3-31.9)	0.24^a^
Mean UDI score (range)	13±4 (4-29)	12±5 (3-39)	0.22^b^
Mean IIQ score (range)	11±3 (6-37)	10±4 (5-42)	0.14^b^
Concomitant surgeries			
TAH	22 (41.5%)		
TAH+BSO	31 (58.5%)	84 (100%)	< 0.01^c^
Cystocele repair	8 (15.0%)	17 (20.2%)	0.17^c^
Rectocele repair	17 (32.0%)	31 (36.9%)	0.13^c^
Perineorrhaphy	18 (33.9%)	34 (40.4%)	0.07^c^
Follow up time	16.3±3.87 (11-23)	16.7±3.04 (10-22)	0.23^a^

Data are presented as mean ± SD

a : Mann-Whitney U-test,

b : Wilcoxon matched pairs test,

c : Fisher’s exact test

No significant major perioperative or early postoperative complication was recorded. The average hospital stay was 3 (2-6) days. Estimated blood loss ranged from 150 to 300 mL. No blood transfusion was required.

The mean follow-up time for the cohort was 16.5 ± 3.45 (range, 10-23) months. Table-II summarizes the preoperative and postoperative findings of the patients. During the follow-up period, in group 1, three patients (%5.6) had a stage-1 cystocele and one (1.8%) a stage 1 rectocele; in group 2, four patients (7.5%) had a stage-1 cystocele and four (7.5%) a stage 1 rectocele. Five months after the surgery, one patient in postmenopausal group developed mesh erosion and underwent excision of the mesh.

**Table-II:** Preoperative and postoperative findings in the patients.

	**Group 1 Premenopausal (n=53)**			**Group 2 Postmenopausal (n=84)**			

	Preop	Postop	p*	Preop	Postop	p*	P**
Mean IIQ-7 score	11±3	5.6± 1.2	<0.01^c^	10±4	2.8±1.8	<0.01^c^	0.03^d^
Mean UDI-6 score	13±4	7.0±1.8	<0.01^c^	12±5	4.2±1.7	<0.01^c^	0.02^d^
SUI	none	15		none	6		< 0.01^b^
Anatomical findings							
Cystocele stage 1		3			4		
Cystocele stage 2	8			17			
Rectocele stage 1		1			4		
Rectocele stage 2	17			31			
Apical prolapse stage 3	48	none		78	none		
Apical prolapse stage 4	5	none		6	none		

SUI: Stress urinary incontinence, UDI-6: Urinary Distress Inventory 6, IIQ-7: Incontinence Impact Questionnaire 7

* Intragroup analysis,

** Intergroup analysis

a: McNemar’s test

b : Fisher’s exact test

c : Wilcoxon matched pairs test

d : Mann-Whitney U-test

Postoperatively, there was no significant difference between groups regarding the surgical effectiveness for POP, but *de novo* SUI was seen in 15 of 53 group 1 patients (28.3%) and in 6 of 84 Group-II patients (7.1%). The stress test was positive in all incontinent patients. Four of the patients (26.6%) in Group-I had resolution of their symptoms with pelvic floor exercise by 6 months, and eleven (73.4%) had a sling performed as an interval procedure after the prolapse surgery. In group 2, all six patients underwent sling operations.

Anatomical recurrence (POPQ ≥ stage 2) was not detected during the follow-up period in either group. Functional outcomes were assessed by comparing the pre- and postoperative IIQ-7 and UDI-6 scores. A statistically significant improvement was noted in various pelvic symptoms and quality of life indexes. Both the intragroup and intergroup differences were significant (Table-II).

## DISCUSSION

In this study, we found that 15.3% (21/137) of the all participants who underwent ASC without incontinence surgery developed *de novo* SUI. These results are consistent with some studies, while others reported higher *de novo* SUI rates (18-20). According to the group analysis, SUI was seen in 15 of 53 (28.3%) Group-1 patients and in 6 of 84 (7.1%) Group-II patients.

Costantini et al. conducted an 8-year follow-up of 66 continent patients who underwent abdominal pelvic organ prolapse repair with or without an anti-incontinence procedure. They reported that 29% of the patients were incontinent after surgery without an anti-incontinence procedure compared with 16% among patients who had a prophylactic anti-incontinence procedure.^18^ Elser et al. described a *denovo* rate of either stress or urge incontinence of 13.3% in patients who underwent ASC alone.^19^ In a prospective trial by Brubaker et al., a *de novo* SUI rate of 44.1% was reported in continent patients who underwent ASC alone (20). We consider these different *de novo* SUI rates to be related to the different follow-up times. In this study, the follow-up time for the cohort was 16.5±3.45 months. This relatively short follow-up time may be a cause of the lower *de novo* SUI rates seen in our study.

The authors of the CARE trial recommended to perform a Burch colposuspension in all women without stress incontinence who are undergoing abdominal sacrocolpopexy for prolapse to reduce postoperative symptoms of stress incontinence. CARE trial founded a higher-than-expected rate of *de novo* SUI in patients undergoing ASC and showed that the addition of the Burch procedure in this group of patients significantly reduced the rate of SUI postoperatively.^20^ However, the addition of an incontinence procedure at the time of POP surgery also increases the risk of developing new-onset urgency or detrusor over activity, as reported in 18-27% of women undergoing Burch urethropexy.^21^,^22^ According to Mallett et al., women undergoing stress incontinence surgery who did not experience resolution of their urgency or frequency were dissatisfied with the surgical outcome, regardless of whether they were counseled preoperatively that the surgery was intended to affect only the stress leakage.^23^

Because of these inconsistent findings, to date, there is no consensus to prevent *de novo* SUI after apical prolapse surgery. Some surgeons perform anti-incontinence surgery in all women undergoing apical repair. Alternatively, some surgeons only perform a prophylactic anti-incontinence procedure if a patient demonstrates SUI. Still other surgeons offer treatment only if SUI develops postoperatively.

In this study, we did not perform anti-incontinence surgery, because all patients were continent preoperatively. The incidence of *de novo* SUI after ASC was 15.3%. Thus, if we had performed a Burch urethropexy in all patients undergoing ASC, an unindicated Burch procedure would have been performed in more than 80% of patients undergoing ASC, subjecting them to unnecessary additional complication risks, including *de novo* lower urinary tract symptoms.

We found that premenopausal women had a higher risk of SUI than that of the postmenopausal women after ASC for prolapse (p < 0.01). The risk was 28.3% in premenopausal women and 7.1% in postmenopausal women. Based on the presence of estrogen and progesterone receptors in the urinary tract,^24^-^26^ female urinary incontinence is assumed to be associated with hormonal status and its fluctuations. In premenopausal women, such hormonal fluctuations affect the structure and function of the lower urinary tract tissue, which could cause *de novo* SUI. One of the most prominent hypotheses regarding this is that the surgery performed to remove the uterus compromises blood flow to the ovaries, which could result in reduced production of hormones, leading to earlier ovarian failure and hormonal fluctuations.^27^

### Limitations of the Study

The major limitations of this study are the small sample size and short follow-up period, but its prospective nature, which enabled direct collection of data, enhanced the validity of the study. In addition, the assessor who was responsible for the follow-up assessment was blinded to the patient baseline data to eliminate any possible bias in the study.

To our knowledge, this is the first study to compare premenopausal and postmenopausal women for *de novo* SUI after ASC without anti-incontinence surgery. Our results indicate that the risk of *de novo* SUI in postmenopausal women after ASC is low. However, premenopausal patients have a higher incidence of stress incontinence which affect quality of life. Nevertheless, further studies with longer follow-up periods are essential to confirm these findings.
